# Transition for Adolescents with Autism Spectrum Disorder: South African Parent and Professional Perspectives

**DOI:** 10.3389/fpsyt.2016.00093

**Published:** 2016-06-07

**Authors:** Meagan Meiring, Joseph Seabi, Zaytoon Amod, Adri Vorster, Anwynne Kern

**Affiliations:** ^1^Department of Psychology, University of the Witwatersrand, Johannesburg, South Africa

**Keywords:** autism spectrum disorders, transition, adolescence, adulthood

## Abstract

Adolescents with autism and their families experience a significant increase in the number of challenges encountered when leaving the structure of the formal education system. The purpose of this study was to gain an understanding of the factors parents and professionals regard as important in preparing for transition of adolescents with autism spectrum disorder (ASD) to adulthood, vocational, and residential arrangements. Semistructured interviews were conducted with 14 participants (i.e., 7 parents and 7 professionals) who were involved with adolescents with ASD in Johannesburg, South Africa. The findings revealed that there was a need for advocacy on behalf of learners with ASD transitioning into adult working and living environments. The responses of the participants highlighted needs for curriculum transformation from basic literacy skills to development and teaching of functional self-help and daily living skills. The results also indicated lack of planning and the absence of service facilities for adolescents with autism post-school. There was a general feeling of fear and uncertainty when the participants thought about transition of adolescents with autism and their future. However, there was also a sense of hope and optimism. Transition of adolescents with autism into adulthood is a challenging and stressful time for parents and professionals involved in trying to prepare them. With appropriate attention and support structures, individuals with autism can attain a reasonable quality of life, including residential, employment, and social opportunities.

## Introduction

Autism spectrum disorder (ASD) is becoming more prevalent, and the statistics suggest that there is an increase in the diagnosis of ASD worldwide, in every racial, ethnic, and social group (1). Transition from school to adulthood is a challenging time for many adolescents with various disorders and their families (2–5). This is especially true for adolescents with ASD, when seeking post-secondary and employment opportunities, as emphasized by Taylor and Seltzer (6) “underemployment of individuals with ASD is an international phenomenon.” Alongside increased stress and uncertainty, families with adolescents with ASD experience reduced support from formal education structures and reduced access to appropriate resources required by the individual and the family (3). An adolescent’s social communication deficits frequently result in increased difficulty in young adulthood and “parents and teachers may observe depressed mood, anxiety, heightened levels of inappropriate behavior, and victimization by peers” (7). Although there is a large body of research conducted on autism and higher education, not enough is known about transition of non-verbal or low-functioning adolescents with ASD post-school, and what it entails to prepare for transition into work and living opportunities. There appears to be no research conducted with regard to adolescents with ASD transitioning to adulthood within a South African context. The feelings that accompany such changes and demands are highlighted within the experiences of the parents and professionals.

According to Hendricks and Wehman (8) (p. 77), “transition typically includes completing school, gaining employment, participating in post-secondary education, contributing to a household, participating in the community, and experiencing satisfactory personal and social relationships.” Transition into adulthood is a particularly stressful time for many parents as they face increased and unique stressors, such as additional financial burdens, more restrictions in social activities, and heightened parental stress (9, 10). Most parents worry about how much independence their child can attain and how they will cope when they can no longer be available to care for them (3). Over and above these, parents also have concerns about behavior and social interactions and communication (11). Taylor and Seltzer (6) pointed out that “although not part of the diagnostic criteria of ASD, maladaptive behaviors are often exhibited by people with ASD … and are a primary source of stress for caregivers.”

It is not only parents of adolescents with ASD who seem to experience challenges but also professionals. Professionals have difficulties in designing and maintaining appropriate programs based on limited research and access to information, and parents report frustration at a lack of information and services of poor quality, as well as high levels of effort required in order to assure a placement for their child in the services provided (8). Professionals are responsible for organizing a team that can prepare and support an individual; however, in the years following formal education, the responsibility falls on the parents to organize a team, on-going learning, and movement into living and working opportunities. As a result of the difficulties experienced by parents and professionals, “many students with ASD leave high school unprepared for adult life at work, in college, or in community living” (7).

The present study was guided by three *research questions*, namely, (1) how is planning for transition being managed and what supports exist during this period of change? (2) What are parent and professional perspectives on an adolescent with autism leaving home and gaining employment? (3) And what thoughts and feelings are experienced by parents and professionals during preparation of adolescents for adulthood?

## Materials and Methods

A qualitative approach using semistructured interviews was used to explore the perspectives of parents and professionals with regard to transition of adolescents with autism. This approach was identified as the most appropriate as the focus of the study was on the exploration of experiences and realities of the perspectives of the participants (12).

Although quantitative research approach encourages sample sizes to be as large as possible in order to ensure generalizability of findings for better validity and quality, the sampling strategy for qualitative research is less concerned with the size of the sample but more with the relevance and adequacy of the sample (13). Therefore, the sample comprised 14 participants, namely, 7 parents and 7 professionals (3 teachers, 1 head of department, 1 principal, 1 skills trainer, and 1 education consultant) working with adolescents between the ages of 16 and 18 years old. Of the 14 participants, only 1 professional was male, and no male parents were represented, which might be due to the fact that the field is feminized, and the participation of more females is in line with stereotypical roles. The ratio of male/female participants creates an interesting bias, specifically with regard to the parents. As evident in Krauss et al. (4), the impact of an adult with autism also focuses more on mothers than fathers. Parent’s ages ranged from 34 to 53 years with a mean age of 47 years 1 month, while professional’s ages ranged from 34 to 79 with a mean age of 49. No further sociodemographic information was collected.

The Johannesburg Hospital School was identified as an appropriate source of participants because it is the only autism-specific ASD school with a senior section for learners up to and including 18 years old in Johannesburg. The school accepts fees on a sliding scale, and some learners are exempt from paying school fees, and thus, the sample does not exclude participants from various socio-economic groups. The learners with autism were mostly within the low-functioning level of the spectrum and non-verbal, or with limited verbal abilities and independence. The adolescents were enrolled in a special needs education setting because of limited access to mainstream curriculum schools and their barriers to learning. The school comprised small classes with a ratio of seven learners to two adults in each class, and each learner worked according to an Individual Education Plans (IEPs) designed by the teacher.

The qualitative data source was conversational data (14) during the interaction between the participants and the first author, which were recorded and transcribed. The interviews took between 45 and 60 min. In order to confirm that saturation of data had been achieved, the authors constructed a saturation grid, wherein major topics were listed on the vertical and interviews to be conducted were listed on the horizontal in line with the guidelines provided by Brod et al. (15).

The data were transcribed and then analyzed using thematic content analysis, based on themes gained through the literature review, as well as remaining open to the idea that data gathered at times generated new insights and themes (12). Common and recurrent themes in the text were identified by creating a list of categories that reflected the major themes. The identified themes were then coded and categorized, and this was followed by analysis and interpretation of the participants’ perceptions (16). Due to the interest in the meaning of transition through the perspectives of professionals and parents, the analytic process often progressed from descriptive themes summarized to interpreting the significance of patterns and their broader meanings and implications (17).

The focus of analysis was on the research question themes pertaining to what plans were being made to prepare adolescents with ASD for transition to adulthood, what supports exist to assist during this transition, and what were the feelings and thoughts of the participants with regard to transition. An interpretation and integration of these was conducted, and conclusions were drawn. The findings from the study are presented together with the discussion.

This study had multiple ethical considerations. First, permission was obtained from the University of the Witwatersrand (Non-Medical Ethics Committee) prior to commencement of the study, and the ethical protocol number allocated was MEDP/12/006 IH. Second, permission to conduct the study was obtained from the participating school. Because the identified school is registered under the Gauteng Department of Education, permission to access the school and to conduct the study was also requested from the relevant department. Finally, permission to conduct research was requested from the potential participants, and only those who agreed to participate were interviewed by the first author.

## Results and Discussion

The results are presented according to the four major themes, namely, curriculum reforms perceived as necessary for successful transition into adulthood; feelings experienced when thinking about transition; support and planning during transition process; and residential and employment opportunities post-school.

### Curriculum Reforms for Successful Transition into Adulthood

The majority of the participants shared a general sense that daily living skills are much more important than knowledge of basic literacy, such as ability to calculate and knowledge of color, as illustrated by the following excerpts:

The things they require to get through their lives are not a *Matric certificate, they need something else (parent 1)

*Within a South African education context, a “Matric certificate” is equivalent to “Grade 12” or a final year of secondary mainstream or formal schooling.

To put it in a nutshell I would say functional learning, … learn to manage their own autism, manage their own behaviour, manage their own learning, so that they can take responsibility for themselves (professional 3)

The school is adapting some of the things in the national curriculum, but we made them relevant to the learners that we teach in terms of content as well as in terms of delivery (professional 4)

The change in focus from academic to functional skills taught within school and home settings appears to be consistent with previous studies (7, 18), which is believed to promote the likelihood of a successful transition. Learners with autism were perceived in the present study to need a different curriculum, due to a different learning style and more relevant, individual, and meaningful content, such as communication, independence, coping with change, vocational skills, and engaging in a community. Functional learning, coping in society and work contexts, emotional management, leisure possibilities, and the characteristics of the triad of impairments were expressed as skills required by individuals with ASD. Transition-focused education needs to take place before leaving school and needs to be a collaborative and active process (7). Consistent with the IDEA (1990) proposals in the USA and South African National Association for Specialised Education (SANASE) (2010) discussions in South Africa, participants in this study also felt that an adjusted curriculum was required because learners with autism would not cope with the mainstream curriculum. In 2001, the White Paper 6 on Special Needs Education was introduced in South Africa, restructuring the education system in order to remove barriers to learning and include children with disabilities into the mainstream education environment. According to this new framework, learners with ASD should be included into mainstream schools, but there is little research on the feasibility and practical implementation of this (19). Education White Paper 6 further suggests that the curriculum needs to be made more flexible so that it is accessible to all learners, irrespective of their needs. Thus, the school implements IEPs focused on goals for future readiness of adulthood. Indeed, Kardos and White (20) assert that transition services are currently not addressing all areas of need of learners in all life areas, including community and personal and social relationships.

### Feelings Experienced when Thinking about Transition

The results indicate a general feeling of fear and uncertainty, when the participants think about transition of adolescents with autism and their future. There is however a sense of hope and optimism that cannot be overlooked, as participants feel that the work that is currently being done with these adolescents is a step in the right direction.

It’s terrifying (laugh) it’s very scary … when they’re little its forgiving, they’re cute and they can do strange things and its forgiving, now that he’s big and the future is out there, its daunting … the scary thing is that we know we are not here forever, so I need to prepare him so that in some way he’s independent, or in a home or whatever his future is, it needs to be put into place a lot sooner than I would like to do it or think about it because the future, I mean I could be dead tomorrow (professional 5)

I find it the scariest thing to even contemplate, I just think, what the hell is going to happen to my son? that is frightening …. I just think that in time that he will, in time I will also get myself there (parent 2)

It’s very exciting, to see our learners transition, because some of them are, they are transitioning, but it’s nerve-wrecking. I am so scared (professional 6)

It’s fearsome sometimes, I am afraid, but it’s interesting also, noticing certain differences and similarities with the normal teenagers, and I think you know that is what gives me strength, to see him maturing from a young child to being a teenager, it makes me think there is a hope somewhere (Parent 4)

The predominant feeling is fear, concern, and terror (58%); however, a substantive proportion of participants (42%) felt a sense of hope and optimism. Both these responses were given by equal amounts of parents and professionals, with some explaining an experience of both fear and optimism. Some participants explained feelings of anger and frustration, mostly feeling that not enough was being done, or that all the work put in would come to nothing after school. Other feelings suggested included confusion, helplessness, unpreparedness, and being tired and having less energy to focus and put things into place. These findings correlate with the literature in that unique stressors are faced, alongside concerns about support opportunities, maladaptive behavior, and communication-deficit impacts (3, 6, 9–11). Some parents also mentioned more positive feelings alongside hope and optimism, such as determination, excitement, and interest, in seeing the adolescent mature.

Blacher (2) (p. 177) states that “the future, when parents seriously begin to think about it, approaches with a barrage of challenges.” This statement is observed to be true when participants begin to explain their thoughts and feelings with regard to the learner’s period of transition out of the formal school structure.

### Support and Planning during Transition Process

The results revealed that the majority of the participants did not have a plan for their children with autism post-school. In addition, over 50% of participants indicated that there was no support for adults with autism. Some of them were planning to encourage the schools to keep the individual with autism as long as possible, thereby indicating that no transition plans exist, as illustrated by the following excerpts.

I don’t think there is any support for them after school. It’s basically at 18 they are the parent’s responsibility (professional 6)

It’s a very very scary thought, it’s a very sad situation, to be very honest, there is nobody, after us (parents) there is really nobody (parent 3)

I don’t think we have any (plans); we go on day by day, doing what you think is right for the child (parent 6)

No I don’t know of any. I have ‘googled’, I have looked, it’s only overseas where I see there is so much support systems (parent 6)

Although there is a growing increase in awareness of the needs and support for adolescents and young adults published internationally (3, 7, 10, 20–22), there is limited accessible information and service facilities in South Africa. Families, schools, and services need support in implementing existing knowledge into practical strategies for these individuals. It seems that minimal support exists within South Africa, for adolescents during transition. Jacklin and Stacey (23) conducted a study to determine accessibility to education for children with autism in South Africa and found that only 30% of LSEN (Learners with Special Education Needs) schools in South Africa provide for learners with ASD. Using the standardization of 10 cases of ASD in every 1000, it is estimated that there are currently between 2 and 21% of learners with ASD who are being schooled in South Africa (23).

### Residential and Employment Opportunities Post-School

Participants were asked to reflect on the possibilities of an adolescent with autism leaving home and gaining employment after having left school. The responses have been understood in three areas of interest: their thoughts on moving out, the possible benefits and the obstacles that are perceived to prevent the possibility, and the perspectives on employment possibilities.

The decision with regard to alternate residential possibilities seems to be approached with great difficulty. Participants expressed feelings of fear and concern, as well as uncertainty with regard to available support services. Although benefits of moving out of home were highlighted by participants, many obstacles were perceived to interfere with the possibility, as indicated by the following quotations:

Half of me thinks it’s a brilliant idea for them to move out; one of the biggest concerns is that, I so worry about care and what happens when I’m not there to guard him (parent 5)

To some extent I think it is beneficial, although it will be very tough, both for the parents and the person. You know the parents are used to supporting the person in every step of the way, and letting go can be very traumatic. But on the other hand I can say it’s beneficial because maybe then they have to be responsible for their things (professional 4)

When he’s older and more adult, I think I would have to look at it (parent 6)

These results are consistent with previous studies, which revealed that increased independence from the family was viewed as beneficial for quality of life of the individual and the family. Wehman et al. (7) highlight the importance of independence from family and confidence in making decisions as two pinnacle areas during the transition period. However, the decision and process are difficult and therefore is often delayed (2, 3). Despite some evidence that the benefits of residential placements include decreased stress of constant caregiving, limiting activities, and social isolation, and decreased concerns when parents aged (4), there was some resistance from the participants to have an individual with autism placed in residential care facility. The theoretical understanding of the benefits are thus hindered by the fear and uncertainty felt by family members. Increased awareness of the positive impact on the family is needed, alongside a wide range of possibilities for residential provision. Once again, quality of life is perceived to be possible; however, increased support is required in order to implement the planning.

Compared to the possible benefits, the obstacles were more easily identified by the parents and professionals. The most common obstacles reported by participants included concerns that there is no available support for the learners with autism, as services are generally provided for learners with special needs, but not autism specific. Participants feared that the individual with autism might be misunderstood or taken advantage of. Communication difficulties and the inability to express discomfort or abuse on the part of the individual with autism added to the perceived vulnerability.

I wouldn’t know if someone was hurting him or not, I wouldn’t know if he’s okay or not, and nobody else would understand him, nor have the patience with him (parent 2)

I haven’t heard or seen anything yet that makes me think, this would be a good idea (parent 1)

Participants in the study perceived employment of people with ASD to be an important goal and one which was considered to be readily obtainable. Unlike typically developing individuals, there was a general sense that those with autism are better employees as they are dependable and loyal. Employees with autism were perceived to be valuable employees as they are reliable, trustworthy, honest, methodological, and with limited interest in social distractions. High levels of intensive support would initially be required by adolescents with ASD; however, it needs to be recognized that “youth with ASD have significant untapped potential that has been underappreciated” (7). They are unlikely to “job hop” when compared to their counterparts, as illustrated by the following excerpts:

Employment and residential facilities are extremely needed, especially for people with autism, because they need to survive and need semi-independent set up later on in their life because parents are not going to be with them forever (parent 3)

I think it’s a possibility and I think it’s fair and I think why should they not have employment, I think if it’s reasonable, and I think it’s important. I think everybody wants to be valued in that way (parent 1)

Employ a person with Autism; I think you won’t regret it (professional 5)

Personally, I would think they would be the best person to employ, depending on the work. They are reliable (parent 1)

Given the right support, given the right attitude they can do things that maybe you and me wouldn’t do (professional 4)

Hagner and Cooney (24) suggest options for methods of modification and preparation, which would support communication and structured tasks in the workplace, and overcome some obstacles for people with autism. Wehman et al. (7) also suggest that “challenges faced by youth with ASD on completion of high school could be improved with a transition-focused education,” and make some suggestions with regard to positive practices. An employer would need to be understanding, patient, and accommodating, and a facilitator would need to support the employer and the employee during the transition. Support is required in modifying tasks and environments, aiding communication, and teaching appropriate behavior. In general, respondents agreed that adults with autism would not cope within a fast pace, demanding or high pressure position, and would engage in a task with increased ease and precision if not reliant on social interaction. Taylor and Seltzer (25) caution though that even if employment is gained, the employee is most likely to be underpaid, due to only menial jobs being acquired as a result of the inadequacy of the service system to accommodate for the needs of individuals. There is a need to examine the role of future employers and their training for employees with ASD is required.

### Implications and Limitations of the Study

The results of the present study suggest that for a successful and smooth transition of adolescents with ASD to adulthood, there is a need for adequate and appropriate preparation, which should include provision of supportive structures, as noted in the curriculum reforms required, and the planning perceived as necessary to support the transition process. Suggested support structures would include preparation of the adolescent and involvement in the process of choice making; improved training for professionals; collaboration between school and home interventions and goals; increased awareness within the community; practical implementation techniques; involved teams, adapted curriculum, and relevant IEPs; and more information on relevant options and planning for the period of transition within individual contexts. Families need increased support and understanding in order to better manage this period of stress, as the findings show significant emotional difficulty for parents and professionals. Improved access to information would increase decision making and coping strategies when considering residential and employment possibilities, and prove beneficial for not only the individual but for the family, professionals, and hopefully have a positive impact on the community.

In terms of the limitations, although the present study laid the groundwork for a larger study, the small sample size precludes generalization of the findings to the entire population with ASD. The experiences of fathers were not taken into account, as only mothers accepted the invitation to participate in the study. This study acts as a springboard for much needed further research within South Africa.

## Conclusion

Transition of adolescents with autism into adulthood is a challenging and stressful time for parents and professionals involved in trying to prepare them, mostly due to fear and uncertainty with regard to what the future holds, as well as a sense of not having done enough to support them, as a result of the scarcity of resources and support. The findings of this study are considered relevant within the South African context as they provide insights into the challenges faced by adolescents with autism and support required. Given the continual increase of autism and the normal life expectancy of individuals with autism, increased awareness of the possible implications on society is required. With appropriate attention and support structures, individuals with autism can attain a reasonable quality of life, including residential, employment, and social opportunities. Increased awareness of possibilities within residential, vocational, and adult services are required, and training facilities and universities could play an increasingly important role in preparing professionals to better understand and support adolescents with autism and their families.

## Ethics Statement

After ethical approval was obtained for this study, from the University of the Witwatersrand’s Human Research Ethics Committee (Non-Medical), the first author invited fifteen schools to participate in the study. Only one school agreed to participate in the study. A written consent was thus obtained from the Gauteng Department of Education (GDE) as the school falls under the demarcation of the GDE.

## Author Contributions

MM made substantial contributions to conceptualisation of the study, performed the literature search, designed data collection instruments, funded the study, collected data for the study, cleaned and analysed the data, and drafted and revised the manuscript. JS made substantial contributions to the conceptualization of the study, supervised the implementation of the study and its write-up, analysed the data, drafted and revised the manuscript and made a contribution to its funding. AK, ZA, and AV participated in the design and coordination of the study, critically revised the manuscript for important intellectual content. All authors read and approved the final manuscript.

## Conflict of Interest Statement

The authors declare that the research was conducted in the absence of any commercial or financial relationships that could be construed as a potential conflict of interest.

## References

[1] Centers for Disease Control. Prevalence of Autism Spectrum Disorders – Autism and Developmental Disabilities Monitoring Network. Morbidity and Mortality Weekly Report, Surveillance Summaries (2012). p. 1–19. 22456193

[2] Blacher J. Transition to adulthood: mental retardation, families, and culture. Am J Mental Retard (2001) 106:173–88. 10.1352/0895-8017(2001)106<0173:TTAMRF>2.0.CO;2 11321608

[3] Howlin P Autism and Asperger Syndrome: Preparing for Adulthood (2nd Edition). London; New York: Routledge (2004).

[4] Krauss MW Seltzer MM Jacobson HT. Adults with autism living at home or in non-family settings: positive and negative aspects of residential status. J Intellect Disabil Res (2005) 49:111–24. 10.1111/j.1365-2788.2004.00599.x 15634320

[5] Sterling LD Characteristics associated with presence of depressive symptoms in adults with autism spectrum disorder. J Autism Dev Disord (2008) 38:1011–8. 10.1007/s10803-007-0477-y 17975722

[6] Taylor JL Seltzer MM. Employment and post-secondary educational activities for young adults with autism spectrum disorders during the transition to adulthood. J Autism Dev Disord (2011) 41(5):566–74. 10.1007/s10803-010-1070-3 20640591 PMC3033449

[7] Wehman P Shall C Carr S Targett P West M Cifu G Transition from school to adulthood for youth with ASD: what we know and what we need to know. J Disabil Policy Stud (2014) 25(1):30–40. 10.1177/1044207313518071

[8] Hendricks DR Wehman P Transition from school to adulthood for youth with autism spectrum disorders: review and recommendations. Focus Autism Other Dev Disabl (2009) 24(2):77–88. 10.1177/1088357608329827

[9] Lecavalier L Leone S Wiltz J. The impact of behaviour problems on caregiver stress in young people with autism spectrum disorders. J Intellect Disabil Res (2006) 50(3):172–83. 10.1111/j.1365-2788.2005.00732.x 16430729

[10] Taylor JL The transition out of high school and into adulthood for individuals with autism and their families. Int Rev Res Mental Retard (2009) 38:1–32. 10.1016/S0074-7750(08)38001-X

[11] Seltzer MM Krauss MW Shattuck PT Orsmond G Swe A Lord C. The symptoms of autism spectrum disorders in adolescence and adulthood. J Autism Dev Disord (2003) 33(6):565–81. 10.1023/B:JADD.0000005995.02453.0b 14714927

[12] Haslam SA McGarty C Research Methods and Statistics in Psychology. London: SAGE (2003).

[13] O’Reilly M Parker N Unsatisfactory saturation: a critical exploration of the notion of saturated sample sizes in qualitative research. Qual Res (2012) 13(2):190–7. 10.1177/1468794112446106

[14] Angrosino M Mays de Perez K Rethinking observation: from methods to context. 2nd ed In: Denzin N Lincoln E, editors. Handbook of Qualitative Research. Thousand Oaks, CA: SAGE (2000). p. 637–707.

[15] Brod M Tesler LE Christiansen TL. Qualitative research and content validity: developing best practices based on science and experience. Qual Life Res (2009) 18(9):1263–78. 10.1007/s11136-009-9540-9 19784865

[16] McMillan JH Schumacher S Research in Education. A Conceptual Introduction. 5th ed New York: Longman (2001).

[17] Braun V Clarke V Using thematic analysis in psychology. Qual Res Psychol (2006) 3:77–101. 10.1191/1478088706qp063oa

[18] Holmes DL Autism Through the Lifespan: The Eden Model. Bethesda: Woodbine house (1997).

[19] Roberts JS Autism and Inclusion: Teacher Perspectives on the Mainstreaming of Autistic Students. Master’s thesis, University of Witwatersrand, Johannesburg (2007).

[20] Kardos MR White BP. Evaluation options for secondary transition planning. Am J Occup Ther (2006) 60:333–9. 10.5014/ajot.60.3.333 16776401

[21] Hanish M Postsecondary Transition in Individuals on the Autism Spectrum. (Masters Thesis). Arizona State University (2011). Available from: http://repository.asu.edu/attachments/56352/content/Hanish_asu_0010N_10313.pdf

[22] Whetstone M Browning P Transition: A Frame of Reference. AFCEC Online journal, special issue, October 2002, (2002). Available from: http://www.afcec.org/pubs/journal/vol1/02F_definition1.pdf

[23] Jacklin L Stacey J Autism South Africa: Aut;talk. 16 ed (2010). Available from: http://aut2know.co.za/wp/wp-content/uploads/Issue16-2010-Part-2.pdf

[24] Hagner D Cooney BF “I do that for everybody”: supervising employees with autism. Focus Autism Other Dev Disabl (2005) 20:91–7. 10.1177/10883576050200020501

[25] Taylor JL Seltzer MM. Employment and post-secondary educational activities for young adults with autism spectrum disorders during the transition to adulthood. J Autism Dev Disord (2010) 41:566–74. 10.1007/s10803-010-1070-3 20640591 PMC3033449

